# Lens-specific βA3/A1-conditional knockout mice: Phenotypic characteristics and calpain activation causing protein degradation and insolubilization

**DOI:** 10.1371/journal.pone.0281386

**Published:** 2023-03-29

**Authors:** Roy Joseph, Michael L. Robinson, Laura Lambert, Om P. Srivastava

**Affiliations:** 1 Department of Optometry and Vision Science, University of Alabama at Birmingham, Birmingham, Alabama, United states of America; 2 Department of Biology, Miami University, Oxford, Ohio, United states of America; 3 Department of Genetics, University of Alabama at Birmingham, Birmingham, Alabama, United States of America; Tsinghua University School of Life Sciences, CHINA

## Abstract

βA3/A1-crystallin is a lens structural protein that plays an important role in maintaining lens transparency via interactions with other crystallins. While the function of βA3/A1-crystallin in the retina is well studied, its functions in the lens, other than as a structural protein, remain unclear. In the current study, we generated the lens-specific βA3/A1-crystallin conditional knockout mouse (named βA3/A1ckO) and explored phenotypic changes and the function of the crystallin in the lens. The βA3/A1ckO mice showed congenital cataract at birth and exhibited truncation of lens proteins. Several truncated protein fragments were recovered as a pellet during a low-speed centrifugation (800 rpm, 70 x g) followed by a relatively higher speed centrifugation (5000 rpm, 2744 x g). Mass spectrometric analysis of pellets recovered following the two centrifugations showed that among the fragments with M_r_ < 20 kDa, the majority of these were from β-tubulin, and some from phakinin, αA-crystallin, and calpain-3. Further, we observed that *in vitro* activation of calpain-3 by calcium treatment of the wild-type-lens homogenate resulted in the degradation of calpain-3, αA-crystallin and β-tubulin and insolubilization of these proteins. Based on these results, it was concluded that the activation of calpain 3 resulted in proteolysis of β-tubulin, which disrupted cellular microtubular structure, and caused proteolysis of other lens proteins (αA-crystallin and phakinin). These proteolyzed protein fragments become insoluble, and together with the disruption of microtubular structure, and could be the causative factors in the development of congenital nuclear cataract in βA3/A1cKO mice.

## Introduction

The vertebrate lens has a unique characteristic of maintaining transparency to perform its major function of refraction. To achieve this transparency, the lens expresses very high levels of structural proteins (crystallins) that include α-, β- and γ-crystallins. Among the crystallins, αA- and αB-crystallins (the two subunits of α-crystallin) play a major role in maintaining lens transparency through chaperone activity [1], which keeps crystallins soluble in their native states. The β- and γ-crystallin genes constitute a superfamily derived from duplication of a common ancestral gene [2]. β-crystallins are composed of acidic and basic oligomeric β-crystallin species whereas γ-crystallins are monomers. The structurally similar β- and γ−crystallins are composed of two similar domains, connected by a short peptide, and each domain is composed of two motifs. Each motifs folds into a distinctive Greek key pattern, a structure required for maintaining lens transparency and refractive power [3,4]. Likewise, the interactions among α-, β- and γ-crystallins are important for maintaining lens transparency [4].

Among acidic and basic β-crystallins, the basic group contains both N- and C-terminal extensions whereas acidic group contains only the N-terminal extension. The acidic βA1-crystallin gene (Cryba1) encodes both βA3- and βA1-crystallins from a single mRNA using alternate translation initiation sites; βA3-crystallin is 17 amino acids shorter than βA1-crystallin. Non-structural functions of βA3/A1-crystallin in the lens remain unclear [3], but β-A3/A1-crystallin possesses protease activity [5–7]. Mutations in CRYBA1 are associated with congenital cataract development [8–10], and one of the most commonly occurring mutation is the βA3ΔG91 mutation [10,11]. Most of the identified βA3 cataract-inducing mutations disrupt crystallin’s native structure resulting in the loss of structural stability, solubility, and ability to oligomerize with other crystallins. These alterations in β-crystallin structure result in protein precipitation that leads to cataract development. However, the molecular mechanism about how the altered structure-function defects of βA3 -crystallin result in cataract development is not fully understood.

βΑ3/A1 (a lysosomal-resident protein in retina [12] is also expressed in retinal astrocytes, retinal pigment epithelial cells (RPE), and retinal ganglion cells [13]. βΑ3/A1 conditional knockout has been shown to impair phagosome degradation in the RPE [14]. Studies in the Cryba1-mutant (Nuc1) in a rat- and genetically modified mouse models have shown that βΑ3/A1 regulates multiple cellular processes in RPE by modulating specific autophagic signaling pathways mediated by V-ATPase (a proton pump). βA3/A1 regulates the ability of V-ATPase to maintain the acidic pH of lysosomes [14]. Presently, it is not known if the lysosomal function of βΑ3/A1 as characterized in the retina, plays a similar role in the autophagic pathway of the lens.

To study the functions of βA3/A1 crystallin in the lens, we previously generated a total CRYβA3/A1 knockout (named βA3KO) mouse [15]. These KO mice were born with nuclear cataract and the cataractous lenses retained their fetal vasculature, a condition caused by failure of the hyaloid artery to undergo normal regression. Further molecular characterization of βA3/A1KO lenses revealed accumulation of autophagic cargo including organelles due to a defective autophagy. In addition, these lenses exhibited aggregation and degradation of cytoplasmic proteins and increased calpain-3 proteolytic activity. Thus, in the lenses of total βA3/A1KO mice, the lysosomal/autophagic systems as well as the calcium-dependent calpain protease functions were altered. To further understand the sequence of events leading to congenital nuclear cataracts in the absence of βA3/A1 function in the lens, we have generated a lens-specific βA3/A1 conditional knockout (named βA3cKO) and determined its phenotypic characteristics and calpain activation.

In this study, we used lenses of the lens-specific βA3cKO- and control mice to: (i) isolate and identify the protein fragments that were present in the insoluble fraction in βA3cKO lenses by mass spectrometry, (ii) confirmed the accelerated proteolysis of phakinin, αA crystallin, calpain-3 and β-tubulin in βA3cKO lenses, (iii) confirmed that calpain-3 activation in wild-type (WT) lens homogenates by calcium treatment resulted in degradation of calpain-3, αA-crystallin and β-tubulin, followed by accelerated insolubilization of their fragmented proteins. Based on these results, we concluded that the disruption of microtubules, and insoluble fragments of lens proteins produced by activated calpain-3 lens might be major causative factors in the development of nuclear cataracts in βA3/A1cKO mice.

Increased accumulation of degraded or damaged proteins, as seen in βA3/A1cKO cataracts represent a common feature in several age-related diseases such as macular degeneration and Alzheimer’s diseases. Additionally, the truncation or mutation in the C-terminal extension of α-crystallin results in myopathies [16,17]. In these diseases, the aggregation and precipitation of proteins is the final step in the development of the disease [18–21]. Although lens crystallins are long-lived proteins with very little turnover [20–22], extensive truncation of lens α-, β-, and γ-crystallins in aging and cataractous human lenses occur [17,23–25]. Our previous reports and those of others have shown that the crystallin fragments not only undergo insolubilization but also form aggregates *in vitro* in human lenses [26–28]. Crystallin- peptides generated *in vivo* also interact with intact crystallins to enhance their aggregation and cross-linking [26,27]. Taken together, these results show that crystallin fragments play an important role in cataract development. Therefore, the observation of the present study of calpain-induced proteolysis of lens proteins might play a major role in congenital cataract development.

## Materials and methods

### Ethics statement

All animal experiments were performed in accordance with protocols approved by the Institutional Animal Care and Use Committee (IACUC) of the University of Alabama at Birmingham (Protocol no. 130208393). Mice were housed in pathogen-free facilities at the University of Alabama at Birmingham. Mice were euthanized by inhalation of CO2, which is the standard method recommended to us for use with mice by the UAB animal care facility. All precautions were taken to minimize of alleviation of suffering and pain to animals during tails snipping for genotyping and during anesthesia injection.

### Generation of βA3-conditional mouse model

The *Cryba1* locus was targeted with the insertion of an FRT-site-flanked selection cassette in intron 3 followed by a loxP site-flanked (floxed) exon 4 as previously described [15]. To delete exon 4 of the of the *Cryba1* gene only in the lens, homozygous floxed mice (βA3^fl/fl^) were crossed with MLR10 mice that express Cre recombinase specifically in the developing lens [29]. Heterozygous floxed mice, positive for MLR10 transgene, were backcrossed to βA3^fl/fl^ mice to obtain MLR10 (Cre) hemizygous mice that were homozygous for the foxed allele. These mice (MLR10; βA3^fl/fi^) are referred as the *Cryba1* conditional knock out or simply the βA3cKO mice. Genotyping of animals was done as described earlier [15]. The primers used for the genotyping are:

Forward primer: 5’-CTGATTTTTGGCAGGTTGAC-3’,

Reverse primer: 5’-TTTCCAGGATGAACTGTTGC-3’ (for the presence of Flp),

Forward primer: 5’-CCTGTTTTGCACGTTCACCG-3’, Reverse primer: 5’- ATGCTTCTGTCCGTTTGCCG -3’ (for the MLR10 Cre transgene),

Forward primer: GGCAGCTGGATTGGTTATG Reverse primer: CCTCCAGCCCCACTAGGGTT (to screen for the deletion of Cryba1 exon 4).

### Quantitative-PCR analysis of lenses of βA3cKO vs. wild type mice

RNA was extracted from lenses of wild type and βA3cKO mice (n = 3), and the cDNA were prepared according to the manufacturer’s protocol (BioRad, Hercules, CA). Real-time PCR quantification was performed using the Bio-Rad iCycler iQ system (Bio-Rad, Hercules, CA). Real-time quantitative PCR (qPCR) was performed using 96-well reaction plate based on our earlier published method [30], using the following primers. βA3 Fwd- 5’-TGGAGTGTGGCGCAATCATA-3’, βA3 Rev- 5’-GAAGTCTGGGCGTGAGATCC-3’. GAPDH Fwd.- 5’ AGGAGAGTGTTTCCTCGTCC. GAPDH Fwd—5′- AGGAGAGTGTTTCCTCGTCC-3’

GAPDH Rev.- 5′-TGCCGTGAGTGGAGTCATAC -3′. The Annealing temperature was 55°C for both the primers.

### Preparation of lens proteins

A total of 4–6 lenses were extracted from wild type (WT), heterozygous (HET) and βA3cKO mice and homogenized in ice-cold buffer A (50 mM Tris-HCl, pH 7.5, 100 mM NaCl containing protease inhibitor cocktail [Roche Diagnostic, Indianapolis, IN]). To isolate crude nuclear and higher molecular weight (M_r_) protein aggregates, lens homogenates were first centrifuged at 800 rpm (70 x g) for 15 minutes at 5°C. The insoluble fraction (pellet) was dissolved in the above buffer A containing 8M urea and the soluble protein fractions were transferred to a new tube and centrifuged again at 5000 rpm for 15 min at 5°C. The supernatant (soluble fraction) was separated, and the insoluble pellet was dissolved again in buffer A containing 8M urea. Equal volumes of the three fractions (buffer A soluble [recovered after 800 rpm centrifugation], the pellet recovered after 800 rpm centrifugation, and pellet recovered after 5000 rpm (2744 x g) centrifugation were analyzed in triplicate by SDS-PAGE using a 12% polyacrylamide gels (BioRad, Hercules, CA). The fragments of proteins with M_r_ lower than 20 kDa, were excised from the gel, and subjected to mass spectrometric analysis as described below.

### Mass spectrometric analysis

The mass spectrometric analyses of fragments of lens proteins from one-month-old wild type and βA3cKO mice were carried out according to the earlier published procedure [15] at the Targeted Metabolomics and Proteomics Laboratory (TMPL) of University of Alabama at Birmingham. To prevent any unwanted proteolysis in lens homogenates prior to SDS-PAGE, we used protease inhibitors as described above. Briefly, the desired SDS-gel bands with M_r_ > 20 kDa were excised and trypsin-digested, and the tryptic peptides were extracted twice for 15 min from the gel pieces using a 1:1 mixture of 5% formic acid and 50% aqueous acetonitrile. Extracts were pooled and evaporated to dryness. The samples were then suspended in 20 μl of 0.1% formic acid and subjected to mass spectrometric analysis. The AB SCIEX 5600 Triple-TOF mass spectrometer (AB-Sciex, Toronto, Canada) was used to analyze the protein digest. Eluted peptides were subjected to a time-of-flight survey scan from 400–1250 m/z to determine the top twenty most intense ions for MS/MS analysis. Product ion time-of-flight scans at 50 msec were carried out to obtain the tandem mass spectra of the selected parent ions over the range from m/z 400–1500. Spectra were centroded and de-isotoped by Analyst software, version TF (Applied Biosystems). In-house MASCOT database searches were carried out against the mouse genome on the NCBInr database.

### Antibodies

The following primary antibodies were used in this study: βA3 crystallin (1:1000 dilution and β-tubulin (1:1000 dilution) [Abcam, Cambridge, MA], β-actin (1:1000 dilution) and GAPDH (1:1000 dilution) [Cell Signaling Technology, Danvers, MA], calpain-3 (1:1000 dilution) [Santacruz Biotechnology, Dallas, TX] and lp82/85 (1:1000) [Sigma-Millipore, St. Louis, MO]. Antibody against phakinin was a generous gift from Dr. Paul Fitzgerald (University of California, Davis), and the polyclonal antibody against the C-terminus of αA-crystallin was generated in house. Secondary antibodies used during western blot analysis were: IRDye 800 CW donkey anti-rabbit IgG (1:20,000 dilution) [Li-Cor Bioscience, Lincoln, NE] and the secondary antibody for immunohistochemistry was Alexafluor 488-conjugated anti-rabbit (1:1000 dilution) [Invitrogen-ThermoFisher Scientific, Atlanta, GA].

### Immunoblotting

Isolated lenses (n = 6) were weighed and homogenized in a radioimmunoassay buffer (RIPA) [Sigma-Millipore, St. Louis, MO] containing protease inhibitor cocktail tablets (Roche Diagnostics, Indianapolis, IN). Protein concentration was determined using a BCA protein assay kit (Pierce Biotechnology, Rockford, IL). Protein preparations (50–100 μg) were electrophoretically separated by SDS-PAGE [31], using 12% polyacrylamide gels (BioRad, Hercules, CA), and transferred to a PVDF membrane using BioRad Turbo System. The blots were blocked with 5% BSA in PBS for 1 h at room temperature, followed by overnight incubation with a desired primary antibody at 4°C. After three washes with PBST (PBS+0.1% Tween 20), the blots were incubated with a secondary antibody. Next, specific protein bands were visualized on a Li-Cor Odyssey system (Li-Cor, Lincoln, NE), and densitometric analysis were performed using image J. The intensity Density (IntDen) is measured by the sum of all the pixel values in the region of interest.

### Slit Lamp microscopy

The mouse was sedated using the Ketamine-Xylazine cocktail and the following dosage was used. 0.1ml per 10 gm. of Body Weight Delivered Dose is Ketamine 100 mg/kg / Xylazine 2mg/kg. Mouse was given an Intraperitoneal injection using 1 ml syringe 23–25-gauge 5/8-inch needle. Mice pupils were dilated with eye drops containing 1% tropicamide and 5% phenylephrine hypochloride. After 15–20 minutes, eyes were examined using a Micron IV slit lamp (Phoenix Research labs, California) at the Vision Science Research Center (VSRC) Core Facility of the University of Alabama at Birmingham.

### Immunohistochemistry

For immunohistochemical studies, mouse eyeballs (n = 4) were fixed in 4% paraformaldehyde in PBS for 24 h and were paraffin-embedded. The 4-μm thick sections were prepared and then de-paraffinized and rehydrated using PBS, followed by heat-induced epitope retrieval in 10 mM sodium citrate and 0.2% Tween 20. The sections were rinsed with water and blocked in 3% (w/v) BSA in PBS for 30 min and incubated overnight with a desired primary polyclonal antibody. The sections were washed with PBS and incubated with anti-rabbit secondary antibodies (Invitrogen) for 1 h at room temperature. Next, the sections were washed with PBS and stained with Hoechst 33342 nuclear stain (1:100 dilution) for 1 min and rinsed with PBS. For the negative controls, the individual primary antibodies were omitted. The sections were mounted with aqueous mounting media and examined with a Nikon confocal microscope, or in some cases, with a Zeiss Axioplan 2 fluorescence microscope equipped with a CCD camera at the Vision Science Research Center-Core facility of the University of Alabama at Birmingham.

### Calpain activity assay

The wild type- and βA3cKO lenses were homogenized in 10 mM HEPES (pH 7.2), 10 mM DTT, 1 mM EDTA, and 1 mM EGTA. The homogenates were centrifuged at 14,000 rpm for 15 min at 5°C, and cytoplasmic supernatant fraction was used to determine calpain activity using a calpain substrate: N-Succinyl-Leu-Leu-Val-Tyr-7-Amido-4-Methylcoumarin (Sigma-Millipore, St. Lois, MO). The luminescence from the substrate cleavage was plotted as a relative Light Unit/ug of protein.

## Results

### Generation of βA3cKO mice and phenotypic characterization

**Fig 1:**
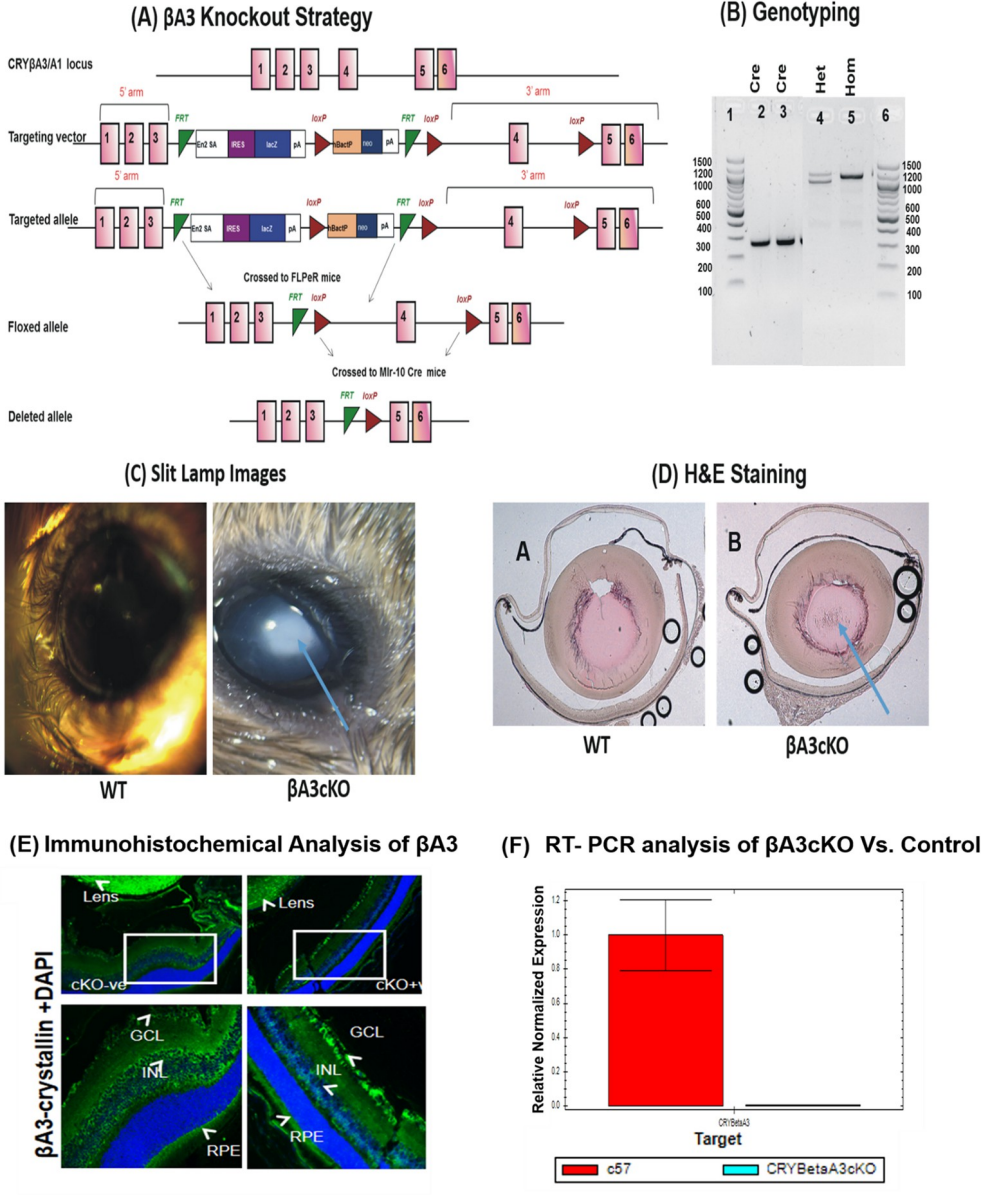
Generation and characterization of lens-specific βA3-crystallin conditional knockout mice. (A) Targeted strategy to generate βA3-crystallin conditional knockout mice (βA3cKO). (B) βA3 mouse genotypic analysis of tail DNA: Lanes 1 and 6: DNA ladder, Lanes 2 and 3 represent presence of the MLR10 transgene, Lane 4: Flip heterozygous expression, and Lane 5: Flip homozygous expression. (C) Micron IV slit-lamp images of 1-month-old wild type lens showing a clear lens (left panel) whereas the βA3cKO mice (1-month-old) lens showing a dense nuclear cataract (identified with a blue arrow). (C) H&E-staining of 1-month-old wild type lens (left panel) and 1-month-old βA3cKO lens (right panel). Note the abnormal nuclear region in βA3cKO lens relative to wild type lens (shown by a blue arrow). (E) One month-old paraffin-embedded eye sections from wild type- and βA3cKO mice immune-stained with anti-βA3-crystallin antibody. The βA3cKO lens exhibited the lack of immunostaining of βA3-crystallin (upper right panel) but not in the wild type lens (upper left panel). The inset of WT lens (cKO-ve) and βA3cKO lens (cKO+ve) are further enlarged below in left and right panels, respectively. In βA3cKO lens (lower right panel), the expression of βA3-crystallin was unaffected in ganglion cells (GLC) and retinal pigment epithelial (RPE) cells and the immunostaining of these cells was comparable to the WT lens (lower left panel). (F) Quantitative-RT PCR analysis of βA3-expression in wild type- and βA3cKO lenses. Note no expression of βA3 in the βA3cKO lens relative to wild type lens. GAPDH was used to normalize the gene expression.

With the objective of understanding the lens-specific functional role of βA3-crystallin, we generated the lens-specific conditional βA3 knockout mice. The overall strategy of construction of βA3-cKO is shown in (Fig 1A). In the construct, which was previously used to generate the total βA3KO [15], the exon 4 of the *Cryba1* gene was flanked by loxP sites (floxed). Exon 4 was selected because this exon encodes amino acids critical for the formation of the Greek key motif 2, important for the structure and dimerization of the βA3-crystallin protein. To generate the lens-specific conditional KO, MLR10 Cre transgenic mice [29] were used. MLR-10 mice express Cre recombinase specifically in the developing lens from about E10.5 onward. Therefore, in the βA3cKO mice, the MLR10 transgene mediated the deletion of *Cryba1* exon 4 only in the lens without affecting *Cryba1* gene expression in other tissues, including retina where *Cryba1* exon 4 is also normally expressed [12–14]. (Fig 1B) shows the mice expressing both lens specific Cre transgene and homozygous for floxed allele. The loss of βA3-crystallin gene expression was confirmed by RT-PCR (Fig 1F). The lenses of βA3cKO mice also developed the congenital nuclear cataract relative to those of wild-type mice as confirmed by the imaging of lenses of 1 month old mice by Micron IV slit lamp (Fig 1C). On hemotoxylin and eosin staining (H&E), the lenses of βA3cKO mice exhibited anomalous nuclear structure (identified by an arrow) relative to those of wild-type mice (Fig 1D). The lack of expression of βA3-crystallin protein from the lenses was confirmed by immunohistochemistry (Fig 1E), and lack of its gene expression by the qRT-PCR analysis (Fig 1F). As shown in the Fig 1E, the expression of βA3-crystallin was mostly abolished only in the lens without affecting its expression in the retina, i.e., in the retinal ganglion cell layer [RGC], and retinal pigment epithelium [RPE]). In addition, the expression of βA3-crystallin in the inner nuclear layer (INL) of the retina on both control and βA3-cKO lenses was also observed (Fig 1E). Although Andley et al. have previously reported the staining for βH (β-high molecular weight fraction) in INL [32], no studies in the past has yet reported specific βA3-crystallin expression in INL. Most importantly, on qRT-PCR, the expression of the βA3-crystallin gene, was not detected in lenses of βA3cKO mice relative to those of wild-type mice (Fig 1F).

### Identification of protein fragments present in insoluble fractions from the βA3-cKO lenses

**Fig 2:**
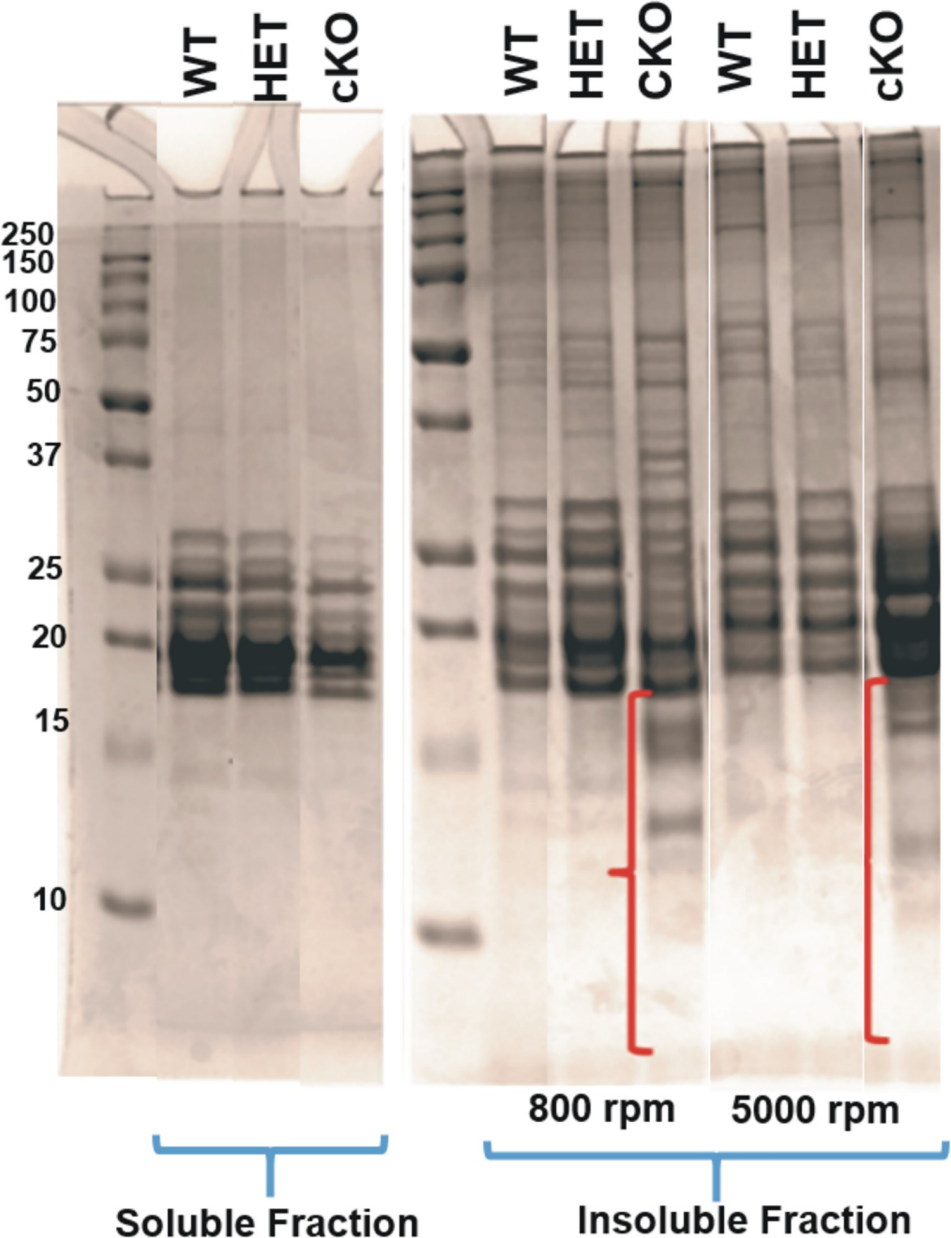
SDS-PAGE analysis of insoluble and soluble protein fractions from lenses of wild type, βA3 heterozygous and βA3cKO mice. SDS-PAGE analysis of insoluble protein fractions recovered after 800 rpm and 5000 rpm centrifugations, and the soluble fractions remaining after the two centrifugations from lenses of wild type, βA3 heterozygous and βA3cKO mice. The protein fragments (< 20 kDa, identified by red brackets) were analyzed by mass spectrometry to identify the parent proteins from which the fragments were derived.

Similar to the lenses of total βA3KO mice [15], the βA3cKO lenses also showed relatively higher amounts of proteins in the water insoluble (WI)-protein fraction compared to the WI-protein fractions from the age-matched control lenses. To further identify proteins and protein fragments that became part of the WI-protein fraction of βA3cKO lenses, the lenses from wild type (WT)-, heterozygous (HET: βA3^fl/wt.^ MLR10Cre ^+/-^) and conditional knockout (βA3cKO: βA3^fl/fl^MLR10^+/-^) mice were separately homogenized (n = 4), and their preparations were subjected to a low centrifugation of 800 rpm to separate the pellet as the crude nuclear fraction. Next, the supernatant recovered after the 800 rpm centrifugation was re-centrifuged at 5000 rpm to isolate the insoluble protein fraction as a pellet, which contained the high molecular weight protein aggregates. Equal amounts of insoluble protein from the protein fractions of βA3cKo and wild type lenses (recovered after 800 rpm and 5000 rpm) and the soluble fractions (recovered after 800 rpm centrifugation) were analyzed in triplicate by SDS- PAGE using a 12% polyacrylamide gel see representative gel in (Fig 2). Both insoluble pellet fractions of βA3cKO lenses recovered after 800- and 5000 rpm centrifugations, respectively, showed increasing numbers of protein fragments (M_r_ < 20 kDa) compared to the protein fragments observed in the insoluble fractions from age-matched WT- and βA3HET lenses [right panels of (Fig 2)]. To identify constituents of protein fragments, present in insoluble fraction, the protein bands with M_r_ < 20 kDa were excised and analyzed by mass spectrometric analysis as described in Materials and Methods. The results showed that the protein fragments found in 800 rpm pellet fraction from HET βA3cKO lenses were 151 compared to 165 fragments found following 5000 rpm centrifugation of the same lenses. After 800 rpm centrifugation, the number of the protein fragments recovered were also higher in βA3cKO lenses compared to those recovered from wild type and HET βA3cKO lenses. However, in contrast, the number of protein fragments recovered after 5000 rpm were greater in number in wild type lenses relative to those from HET βA3cKO and βA3cKO lenses Table 1.

**Table 1:** Identification of protein fragments (>20 kDa) by mass spectrometric method in the pellet recovered following 800 rpm spin and 5000 rpm spin from lenses WT (βA3^fl/fl^ Cre^-/-^), HET (βA3^fl/-^ Cre^-/+^) and cKO [conditional n KO] (βA3^fl/fl^ Cre^-/+^) mice.

Sample	Total number of fragments identified derived from different proteins 800 rpm spin 5000 rpm	
Wild type ((βA3^fl/fl^ Cre^-/-^))	149	200
Heterozygous (HET)	151	165
Conditional KO (cKO)	176	176

The protein fragments present only in the βA3cKO lenses but not in WT and βA3HET lenses were selectively identified by the mass spectrometric analysis. These unique protein fragments of 800 rpm and 5000 rpm pellet fractions were further divided into two groups based on the molecular weights of the parent proteins from which they were derived. The fragments that were derived from the proteins of M_r_ > 25 kDa were included in group I, and the fragments derived from the proteins M_r_ < 25 kDa were included in group II. The protein fragments present only in the βA3cKO lenses after 800 rpm centrifugation are listed in Table 2. Group I included the fragments from a total of eleven proteins, of which the eight fragments were derived from different chains of β-tubulin, suggesting that β-tubulin fragments were derived due to a relatively greater degradation of the protein in the βA3cKO lenses. Group II in Table 2 contained the fragments from 15 different proteins (M_r_ ranging from 8 to 23 kDa) that included a number of mitochondrial proteins, small heat shock proteins and small ubiquitin-related modifier proteins.

**Table 2:** Mass spectrometric identification of protein fragments present in the insoluble fraction of βA3 cKO lenses but not in WT and HET lenses following centrifugation at 800 rpm.

Group I		
Protein description	Counts	Mol. Wt. (kDa)
Cell division cycle 7-related protein kinase (Cdc7)	1	63
Reversed Immunoglobulin superfamily containing leucine-rich repeat protein 2 (Islr2)	1	79
Transcriptional regulator Kaiso (Zbrb3)	1	74
Tubulin beta-1 chain (Tubb1)	1	50
Tubulin beta-2A chain (Tubb2a)	2	50
Tubulin beta-2B chain (Tub2b)	2	50
Tubulin beta-3 chain (Tubb3)	2	50
Tubulin beta-4A chain (Tubb4A)	1	49
Tubulin beta-4B chain (Tubb4b)	1	50
Tubulin beta-5 chain (Tubb5)	2	49
Tubulin beta-6 chain (Tubb6)	1	50

**Group II**		
**Protein description**	**Counts**	**Mol. Wt. (kDa)**
10-Da heat shock protein, mitochondrial (Hspe1)	2	10
Cytochrome c oxidase subunit (NDUFA4)	1	9
Cytochrome somatic (Cycs)	1	11
Guanine nucleotide-binding protein G(I)/G(S)/G(O)subunit gamma-11 (Grig11)	1	8
Guanine nucleotide-binding protein G(T) subn unit gamma-T1 (Gngt1)	1	8
Mitochondrial pyruvate carrier 1 (Mpc1)	1	12
Myosin light chain 6B (Myl6b)	1	20
Programmed cell death protein 5 (Pdcd5)	1	14
Retinal rod rhodopsin-sensitive cGMP 3’,5’-cyclic phosphodiesterase subunit gamma (Pde6g)	1	9
Retinol-binding protein 1 (Rbp1)	1	15
Small nuclear ribonucleoprotein E (Sntpe)	1	11

The protein fragments that existed as an insoluble pellet after 5000 rpm centrifugation in βA3cKO lenses are listed in Table 3. Similar to the insoluble protein fraction recovered after 800-rpm centrifugation, the group I of the 5000-rpm centrifugation fraction also contained a number of beta-tubulin fragments. The fragments of cell division cycle 7-related protein kinase (Cdc7) were also present in the group I from both insoluble fractions recovered after 800 and 5000 centrifugations. The group II of 5000 rpm spun-fraction included fourteen different proteins including small molecular weight mitochondrial proteins such as cytochrome C oxidase. Since the molecular weights of the proteins in group II were < 25 kDa, two possible reasons could be attributed for their presence in the insoluble pellet fractions: (i) increased targeted proteolysis of these proteins that resulted in aggregation and insolubilization of the protein fragments, and/or (ii) these proteins were aggregating along with the protein fragments generated by group I proteins without themselves being subjected to proteolysis. Further, the presence of a striking number of β-tubulin fragments in both 800- and 5000 rpm pellet fractions were seen only in the βA3cKO lenses suggested that: (i) the β-tubulin undergoes accelerated proteolysis in βA3cKO lenses relative to wild-type lenses, and (ii) the proteolyzed fragments of β-tubulin accumulate and form aggregates and become the part of the insoluble protein fraction, and (iii) microtubules disruption occur in βA3cKO lenses relative to WT and heterozygous lenses, which might disrupt cytoskeleton.

**Table 3:** Fragments of proteins present only in the insoluble fraction of βA3 cKO lenses relative to WT and HET lenses following centrifugation at 5000 rpm.

Group I		
Protein description	Counts	Mol. Wt (kDa)
Calpain-3 (Capn3)	1	94
Cell division cycle 7-related protein kinase(Cdc7)	1	63
Centrosomal Protein of 170 KDa protein B (Cep170b)	1	170
CLIP-associating protein 1 (Clasp1)	1	160
Poly(rC)-binding protein 1 (Pcbp1)	1	37
REVERSED Pentatricopeptide repeat-containing protein 1, mitochondrial (Ptcb1)	1	78
Tubulin beta-1 chain (Tubb1)	1	50
Tubulin beta-4A chain (Tubb4a)	1	49

**Group 2**		
**Protein**	**Counts**	**Mol. Wt (kDa)**
AP-2 complex subunit sigma (Ap2s1)	2	17
ATP synthase subunit delta, mitochondrial (Atp5d)	2	17
CDGSH iron-sulfur domain-containing protein (Cisd1)	2	12
Cytochrome b-c1 complex subunit 9b (Uqcr10)	1	7
Cytochrome c oxidase subunit 6B1 (Cox6b1)	1	10
Cytochrome c oxidase subunit 6C (Cox 7a2)	1	9
Cytochrome c oxidase subunit 7A2, mitochondrial (Cox7a2)	1	9
Cytochrome c, somatic (Cycs)	1	11
Myosin light chain 1/3, skeletal muscle isoform (Myl1)	3	21
NADH dehydrogenase [ubiquinone] 1 subunit C2 (Ndufg2)	1	15
Peroxiredoxin-2 (Prdx2)	1	22
Profilin-2 (Pfn2)	1	15
Retinal rod rhodopsin-sensitive cGMP 3’,5’-cyclic phosphodiesterase subunit gamma (Pde6g)	1	9
Translation machinery-associated protein 7 (Tma7)	1	7

**Fig 3:**
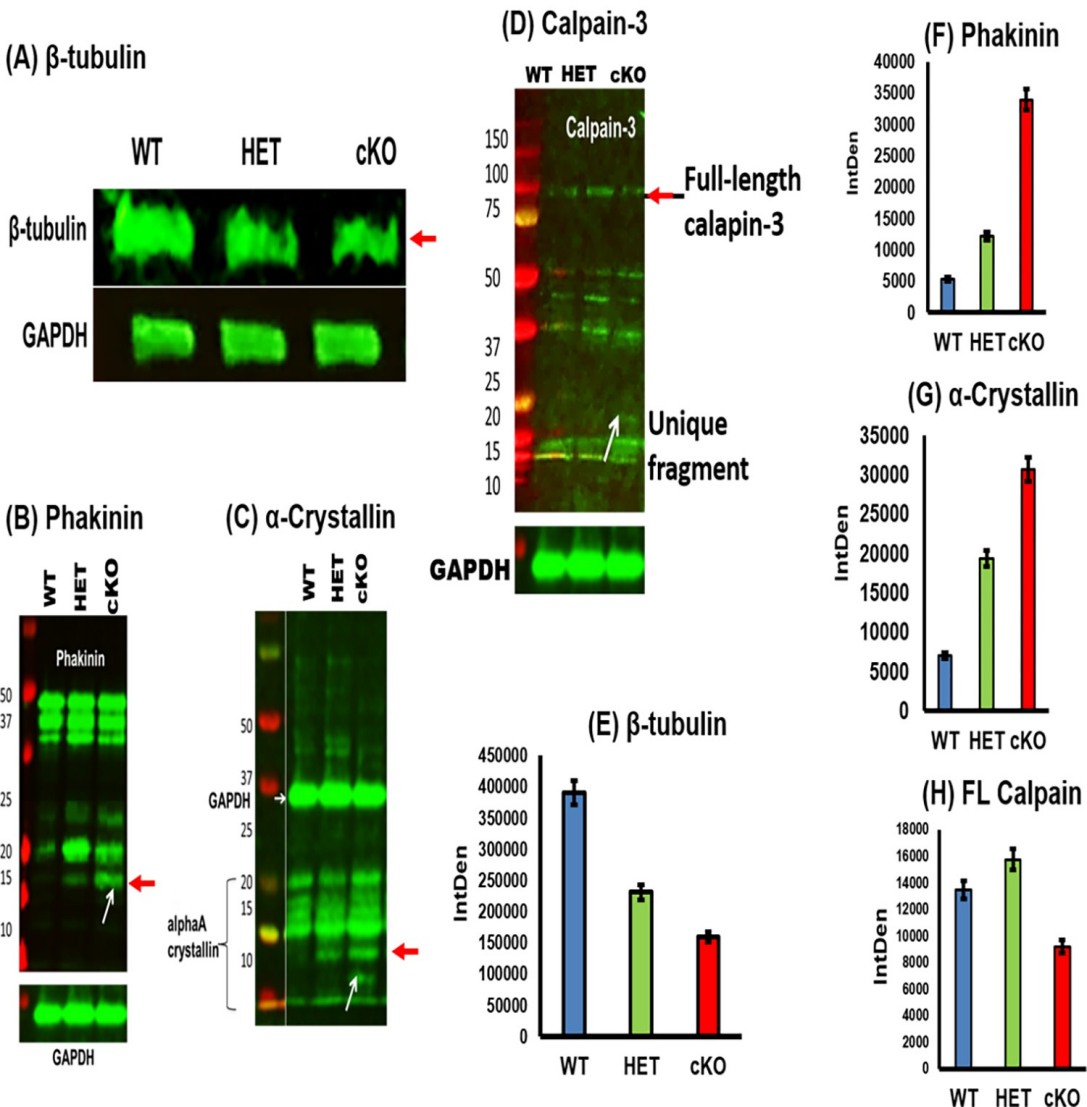
Increase in fragmentation of lens proteins in lenses of βA3cKO relative to wild type and βA3 heterozygous lenses as determined by western blot analyses. **(**A) About 40% decrease was seen in the levels of β-tubulin in βA3cKO lenses relative to wild type and βA3 heterozygous lenses. GAPDH was used as loading control. (B) Increase in fragmentation of phakinin (lane 3, identified with an arrow) in βA3cKO lenses relative to wild type- and heterozygous lenses (lanes 1 and 2) (C) Increase in fragmentation of αA-crystallin in βA3cKO lens (lane 3, identified with an arrow) relative to wild type- and heterozygous lenses (lanes 1 and 2). (D) Increase in fragmentation of calpain-3 in βA3cKO lens (lane 3, identified with an arrow) relative to wild type- and heterozygous lenses (lanes 1 and 2). In (A), (B), (C) and (D), the βA3cKO lenses showed lower levels of full-length and additional fragmented protein bands of β-tubulin, phakinin, α-crystallin, and calpain, respectively relative to lenses from wild type- and heterozygous mice. In (C), an increase in fragmentation of α-A crystallin along with multimers of alpha A crystallin of different sizes were observed on SDS-PAGE analysis. The antibody raised against full-length α-A crystallin was used. βA3cKO lenses showed a decrease in the amount of different sized multimers and increase in the amount of αA-crystallin monomer and proteolyzed fragments, compared to the wild type lenses (Results not shown). The protein fragment bands in the far-right lanes shown by a red arrow of images (A), (B), (C) and (D) were quantified using Image J and are represented as bar graphs in Figs. (E), (F), (G) and (H), respectively.

To confirm that β-tubulin is proteolyzed in βA3cKO lenses, the total lens protein fractions recovered after homogenization of 1-month old control (WT) and age-matched HET βA3cKO and βA3cKO lenses were probed by Western blot using anti-β-tubulin antibody (Fig 3A). The total levels of β-tubulin were substantially reduced by 40% [densitometric analysis, (Fig 3E)] in the βA3cKO lenses compared to the WT lenses when GAPDH loading control was used to confirm the equal amounts of loading of the proteins. Although the anti-β-tubulin antibody used was raised against the full-length protein in this analysis, the results did not show any β-tubulin fragments on Western blot.

In addition to the presence of above-described unique protein fragments in βA3cKO lenses, the comparison of the list of protein fragments and their spectral counts following mass spectrometric analysis of 800 rpm and 5000 rpm pellets showed that the degradations of αA-crystallin, phakinin and filensin were relatively increased in βA3cKO lenses compared to WT and βA3HET lenses Table 4. Western blot analyses with antibodies against phakinin and αA-crystallin were utilized to further confirm the increased degradation of these proteins. As shown in (Fig 3B) the intensity of full-length phakinin band (49 kDa) and its cleaved bands (>25 kDa) were slightly decreased whereas the intensity of fragmented smaller molecular weight bands (<15 kDa, shown by an arrow in (Fig 3B) were relatively increased in the βA3cKO lens fraction (lane 3) compared to the intensity of bands from the fractions of WT and βA3HET lenses. Similarly, when the lens fractions were probed using an anti-αA-C-terminus antibody, the βA3cKO lenses showed an increase in degradation of the full-length protein, and the presence of additional fragments compared to the age-matched WT and βA3HET lenses [(Fig 3C), lane 3)]. Similarly, on western blot analysis with anti-calpain 3-antibody, calpain 3 degradation was also observed in βA3cKO lenses relative to WT and βA3HET lenses [(Fig 3D), shown by an arrow in lane 3]. Together, these findings suggest that the degradation of tubulin, calpain-3, phakinin, and αA-crystallin was relatively increased in βA3cKO lenses relative to βA3HET- and WT-lenses. Fig 3E, 3F, 3G and 3H show the intensity Density (IntDen) measured using image J of the lanes that are indicated by a red arrow in (Fig 3A–3D) respectively. In the (Fig 3E), the pixel intensity for β-tubulin was analyzed, which shows that in the lenses of βA3cKO mice, there was a decreased β-tubulin expression compared to the lenses of HET and wild type mice. In the case of phakinin and α-crystallins, we measured the low molecular weight fragments that were increased as evidence of degradations. Fig 3F and 3G show increased expression of low molecular weight fragments in the lenses of βA3cKO mice compared to the HET and Wild type mice. When full length calpain was measured, there was a decreased in expression of full length calpains (FL calpain) in cKO mice compared to the HET and wild type mice (Fig 3H).

**Table 4:** Spectral counts in αA-crystallin, phakinin and filensin during mass spectrometric analysis.

Proteins	Spectral counts					
	**800 rpm centrifugation**			**5000 rpm centrifugation**		
	WT	HET	cKO	WT	HET	cKO
αA-crystallin	10	16	24	17	21	37
Phakinin	6	1	7	11	5	19
Filensin	4	2	10	13	9	10

### Confirmation of calpain-3 activation in βA3cKO lenses

**Fig 4:**
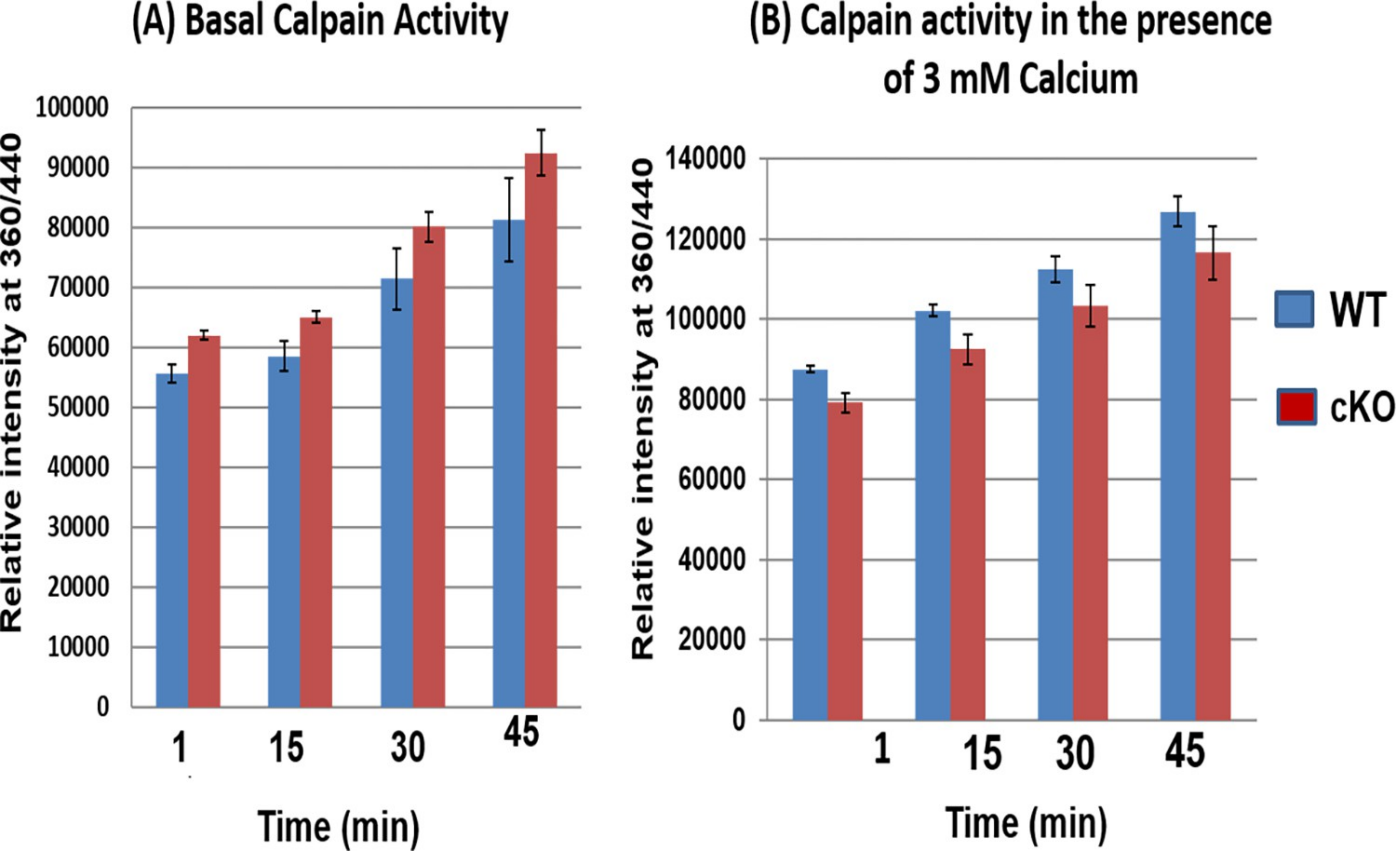
Characterization of levels of calpain-3 activity in βA3cKO wild-type lenses. Calpain activity was determined using N-succinyl-leu-tyr-7-amido-4 methyl coumarin as a substrate. (A) Basal calpain activity in absence of calcium chloride in homogenates of βA3cKO- and wild-type lenses. The βA3cKO lens homogenate exhibited a 10% increase in basal calpain activities compared to the wild-type lenses with increasing time, **(**B) Calpain activity in the presence of 3 mM calcium chloride in homogenates of βA3cKO- and wild-type- lenses. About 10% decrease in calpain-3 activity in the presence of 3 mM Ca^+2^ was observed by the βA3cKO lens homogenate relative wild-type lens homogenate.

**Fig 5:**
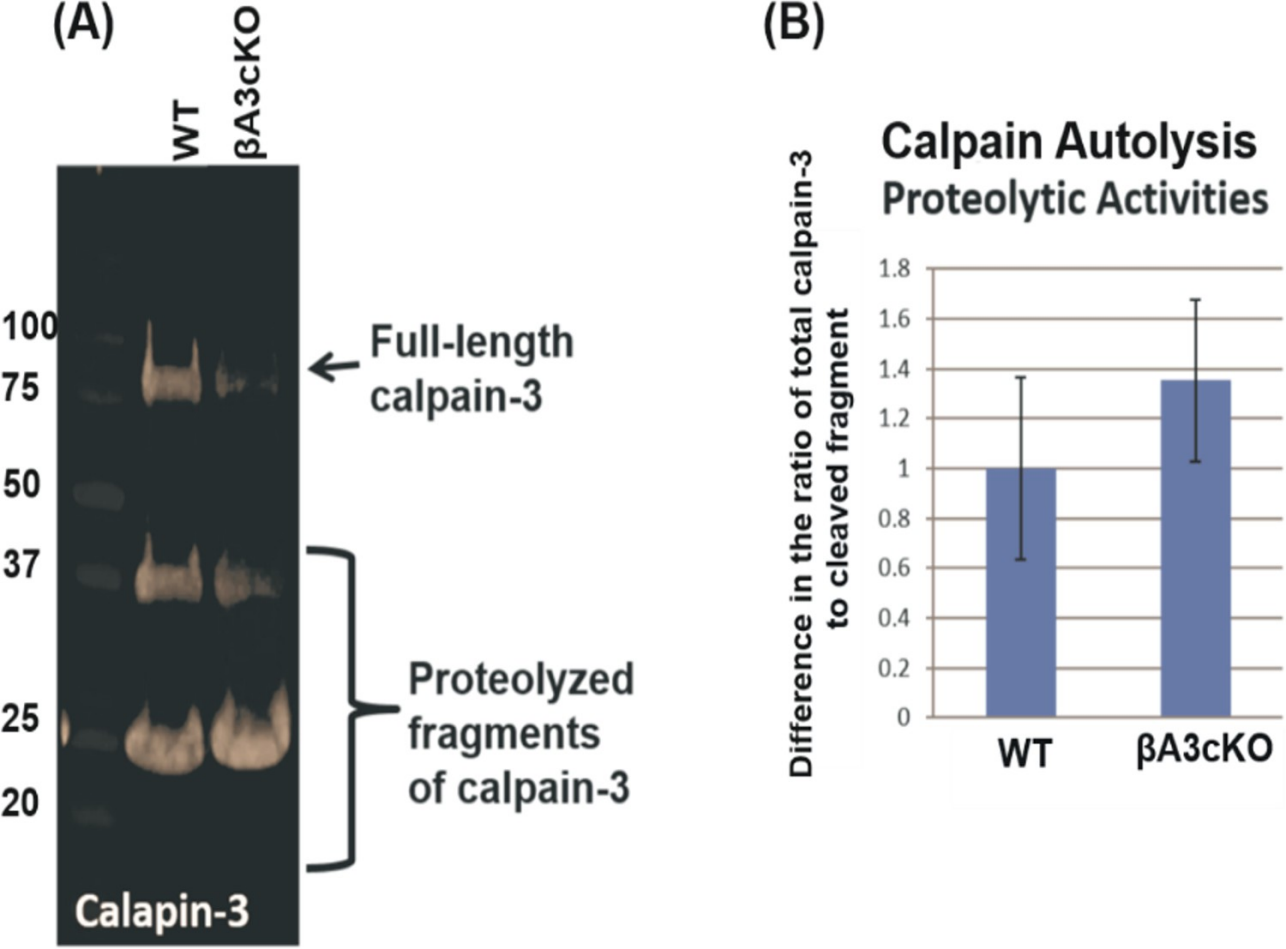
Determination of autolysis of calpain-3 in lens in homogenates of βA3cKO and wild-type lenses by western blot analysis. (A) The Calpain-3 activity was assessed by determining the ratio of total calpain-3 protein (the intensity of full length calpain 3 band + intensity of cleaved fragments) to the amount of uncleaved full-length calpain-3 (intensity of full length calpain-3 band) in homogenates of βA3cKO and wild-type lenses using the western blot analysis. (B) As shown in the Figure, in wild type lenses the ratio of full-length to cleaved fragment was 2.4, whereas in lenses the ratio was decreased to 1.7. This means that relative to wild type lenses, the amount of full-length protein was decreased in βA3cKO lenses, and the amount of cleaved fragment was increased. Overall, the quantification of autolyzed calpain fragments in βA3cKO lenses vs. wild type lenses showed a 35% increase in calpain-3 proteolytic activity in βA3cKO lenses compared to the wild type lenses.

**Fig 6:**
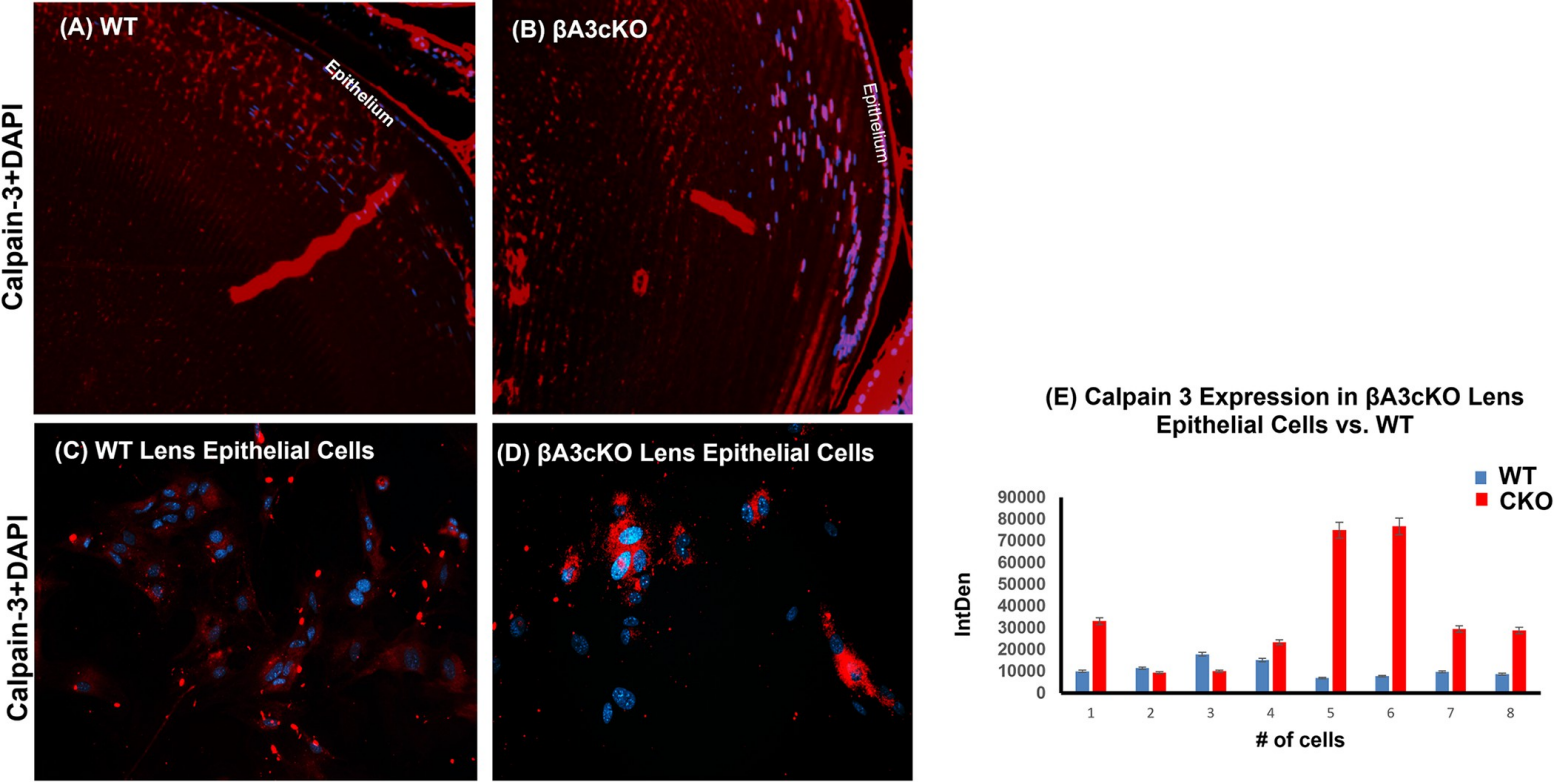
Immuno-localization of calpain -3 in Lenses and cultured lens epithelial cells of βA3cKO and wild-type mouse. (A) and (B) Immunohistochemical localization of calpain-3 in in lenses of βA3cKO and wild type lenses. (C) and (D) Immunocytochemical localization of calpain-3 expression in cultured lens epithelial cells from wild-type lenses (C) and those from βA3cKO lenses (D). (E) Calpain-3 expression was quantified in lens epithelial cells from wild type lenses (C) and βA3cKO lenses (D) using image J. Note and that the immunohistochemical localization showed that calpain-3 was mostly localized in the outer cortical region in the wild type lenses (A), but it was mostly associated with nuclei in the βA3 βA3cKO lenses (B). There was an increased calpain -3 expression in βA3cKO lens epithelial cells compared to control lens epithelial cells (Fig 6E).

Interestingly, in addition to β-tubulin, fragments of calpain-3 were observed only in 5000 rpm spun-pellet fraction of cKO lenses but not in WT- and βA3HET-lenses Table 3. Earlier studies have shown that calpain being associated with cataract development. To further characterize calpain-3 activity in βA3cKO lenses, an *in vitro* calpain activity assay was carried out using N-succinyl-leu-tyr-7-amido-4-methyl coumarin as a substrate. To determine calpain-3 activity, total protein homogenates from 1-month-old WT- and βA3cKO-lenses were incubated with the substrate in the absence of calcium (representing the basal activity of calpain) and in the presence of 3 mM calcium (representing Ca^2+^-induced calpain activation), and the release of products was monitored at 15 min intervals up to 45 min. The βA3cKO lens homogenate exhibited a 10% increase in the basal calpain activity when compared under similar activities of the control lenses with increasing time (Fig 4A). However, despite 20% increase in calpain-3 activity in the presence of 3 mM Ca^+2^ by the βA3cKO lens homogenate relative to the basal calpain activity (Fig 4B) of the control lens homogenate, the calcium-induced activity was about 10% less. The ratio of total calpain-3 to its uncleared calpain-3 in the βA3cKO lenses versus control lenses was determined by Western blot analysis. The bands were analyzed by Image J analysis. As shown in the Fig 5A and 5B, in the WT lenses the ratio of full-length to cleaved fragment was 2.4, whereas in βA3cKO lenses the ratio was decreased to 1.7. This means that relative to WT control lenses, the amount of full-length protein was decreased in βA3cKO lenses, and the amount of cleaved fragment was increased. Overall, the quantification of autolyzed calpain fragments in βA3cKO lenses vs. control lenses showed a 35% increase in calpain-3 proteolytically produced fragments in βA3cKO lenses compared to the calpain-3 in the WT lenses. Additionally, on immunostaining of lenses sections from 1-month old WT- and βA3cKO mice with anti-calpain 3-antibody, the levels of calpain-3 staining in WT lenses was throughout the outer cortex (Fig 6A), but it was localized only in the outer cortex and associated with nuclei in the βA3cKO lenses (Fig 6B). Next, we examined the expression of calpain-3 in lens epithelial cells in culture of WT- and βA3cKO mice (Fig 6C and 6D). Calpain-3 expression in lens epithelial cells were quantified using image J (Fig 6E). There was an increased expression in βA3cKO lens epithelial cells compared to those from wild type lensed (Fig 6E).

### Activation of calpain-3 by elevated calcium levels *in vitro* of WT lens homogenate increases proteolysis of αA-crystallin and β-tubulin and induces insolublization of lens proteins

**Fig 7:**
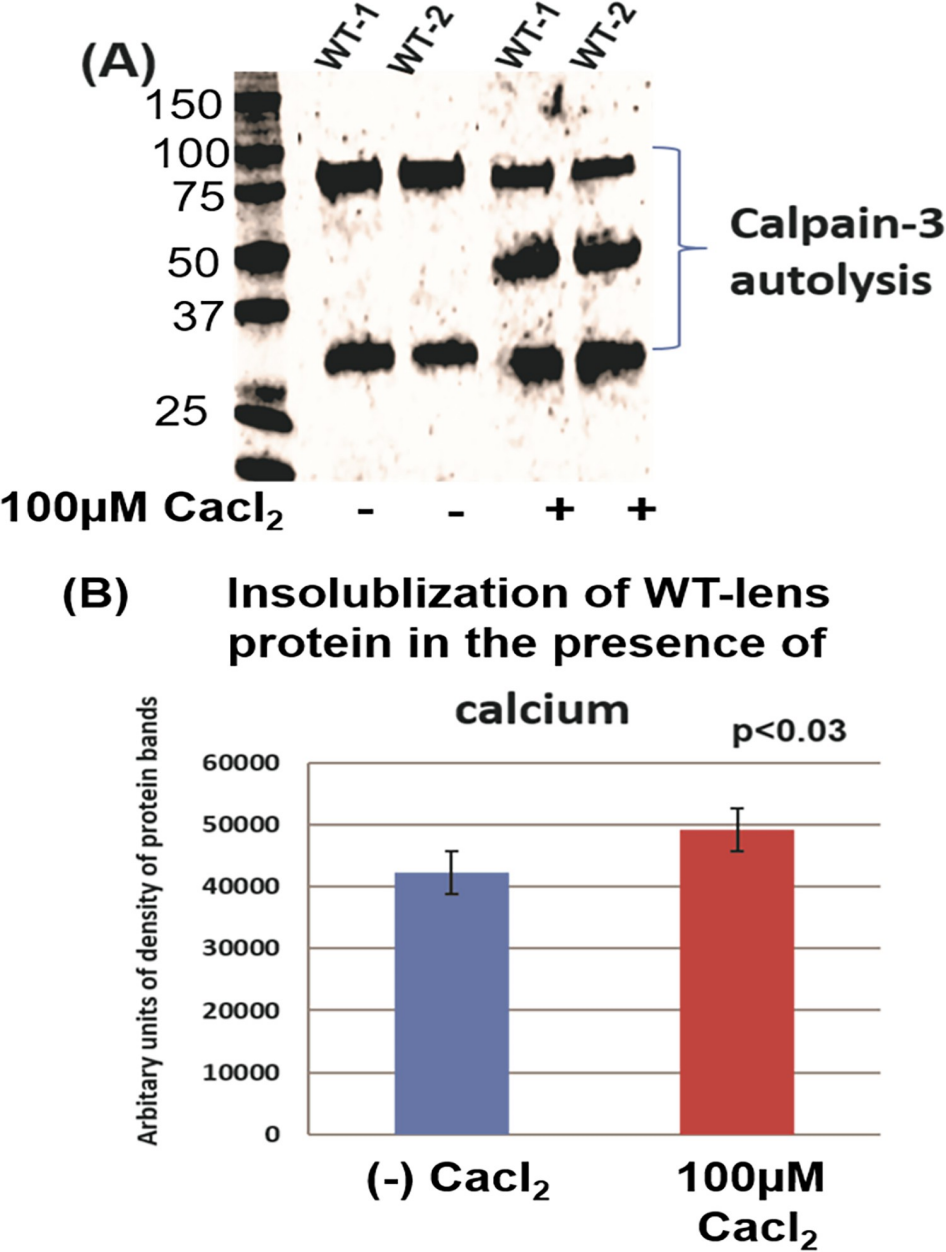
Activation of calpain-3 in homogenate of wild type lenses causes accelerated proteolysis and insolublization of lens proteins. The wild type of lens proteins was homogenized in 10 mM Tris-HCl (pH 7.8) containing 10 mM DTT and 1% SDS and incubated with and without calcium chloride (100 μM) for 2 h at 37°C. (A) Calpain-activation (autolysis) was monitored by western blot using anti-calapin-3 antibody. In the presence of 100 μM calcium, the autolysis of calpain-3 was increased. (B) A greater insolubilization of proteins occurred in the presence of 100 μM calcium than in absence of Ca^+2^. Both Ca^+2^-treated and untreated preparations were centrifuged at 5000 rpm and the concentrations of proteins in the soluble- and insoluble-fractions (pellet) were determined by densitometric analysis of proteins after SDS-PAGE. An accelerated autolysis of calpain in Ca^+2^-treated preparation was accompanied with a relatively higher amounts of insoluble proteins.

**Fig 8:**
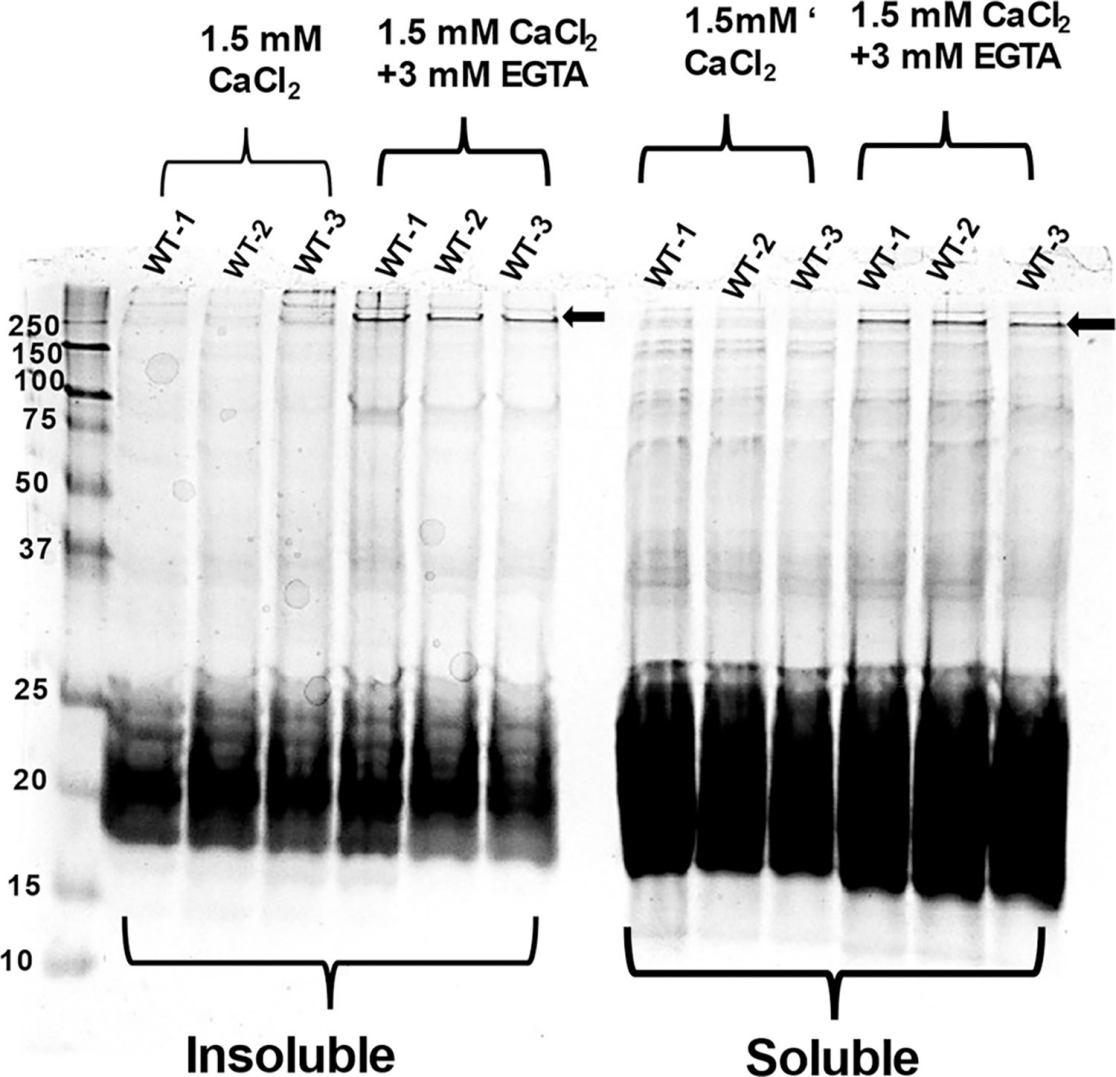
SDS-PAGE analysis of spectrin- degradation in water soluble (WS) and water insoluble protein fractions with addition of 1.5 mM CaCl_2_ and without 3 mM EGTA in wild-type lenses. SDS-PAGE analysis to determine spectrin-degradation in water soluble (WS)- and water insoluble (WI) protein fractions from three wild-type lenses following incubation for 3 h with 1.5 mM CaCl_2_ and with and without 3 mM EGTA. Note a 250 kDa protein band of α-spectrin was intact in the presence of EGTA but was absent in the absence of EGTA, suggesting that calpain proteolyzed the protein in the absence of EGTA. This suggested that calpain was active in proteolyzing α-spectrin in the presence of exogenous CaCl_2_.

Because only calpain-3 is activated at the μM level of calcium concentration, next it was determined whether treating the WT-lens protein homogenate with low calcium (100 μM) could cause: (i) activation of calapain-3, (ii) increase the autolysis of calpain 3, and (iii) induce the insolubilization of lens proteins. In this experiment, 1-month old WT lens protein homogenate was incubated with (at 100 μM Ca^2+^) and without calcium for 3h at 37° C followed by SDS- PAGE analysis to determine autolysis of calpain 3. Equal amounts of calcium-treated and untreated proteins of the homogenates were analyzed by western blot analysis using anti-calpain-3 antibody (Fig 7A), which showed autolysis of calpain 3 occurred but only in the presence of Ca^2+^. To determine the insolubilization of lens proteins, both Ca^+2^-treated and untreated preparations were centrifuged at 5000 rpm and the concentrations of proteins in the soluble- and insoluble-fractions (pellet) were determined by densitometric analysis of proteins after SDS-PAGE (Fig 7B). In addition to the accelerated autolysis of calpain Ca^+2^-treated preparation (Fig 7A), a relatively higher amounts of insoluble proteins (Fig 7B) were observed in the Ca^+2^-treated fraction. However, in the presence of Ca^2+^, neither β-tubulin nor αA-crystallin showed proteolysis. To determine whether activated calpain 3 proteolyzed any other lens proteins, the proteolysis of α-spectrin was determined. The rationale was that α-spectrin is a preferred lens protein substrate for calpain 3, which has been used in past studies as the signature protein substrate for *in vivo* calpain 3 activation and its induced proteolysis [33,34]. As shown in Fig 8, intact α-spectrin (M_r_ 250 kDa) was observed in lens water soluble and water insoluble fractions of wild type lenses after incubation with 150 mM Ca^2+^ plus EGTA whereas it was absent in these two fractions of homogenates incubated with 150 mM Ca^2+^ alone (α-spectrin band of 250 kDa identified by arrow in (Fig 8). In our previous publication, we also observed the degradation of 250 kDa spectrin band (analyzed by its excision and identification by mass spectrometry) in the lenses of total βA3 KO mice [15]. The results suggested that the calpain 3 was active, but it did not proteolyze β-tubulin and α-crystallin in our assay. Cataract development also altered the cytoskeletal structure and therefore, only cataractous lenses showed calpain-induced proteolysis of β-tubulin and α-crystallin.

## Discussion

The βA3/A1 crystallin is structural lens protein and is also localized in the retina. For a variety of reasons, βA3/A1 crystallin is a unique protein for the some of the following reasons: (A) Besides beings a lens structural protein [2], the lens βA3/A1-crystallin expression was also detected in the neural retina (specifically in the astrocytes, ganglion cells and in the retinal pigmented epithelium (RPE) [12–14]. In the retina, the crystallin has been shown to be a lysosomal resident protein and functions as the autophagy modulator [14]. (B) Aggregation properties of a mouse lens recombinant beta A3/A1-crystallin without its N-terminal extension relative to a normal recombinant mouse beta A3/A1-crystallin suggested that the N-terminal arm of the crystallin facilitates dimer formation and is necessary for higher-order associations [35]. Further, the sequence of the connecting peptide is not critical for the association of beta A3/A1-crystallin into dimers and higher order aggregates [36]. (C) On calculation of the ratio of silent to nonsynonymous substitution between orthologous βA3/A1-, βB3-, and other β- and γ-crystallin sequences, the region encoding the two globular domains of the βA3/A1-crystallin sequence is the best conserved during evolution, and much better than the corresponding region of the βB1-, βB3-, or the g-crystallin sequences, and even better than the well-conserved rodent alpha A-crystallin sequence [37]. (D) βA3/A1-crystallin regulates apical polarity and EGFR endocytosis in retinal pigmented epithelial cells [38]. (E). Because of calcium-binding to βB2- and βA3-crystallins, this property has important implications for understanding the calcium-related cataractogenesis and maintenance of ionic homeostasis in the lens [39].

To understand the functions of βA3/A1-crystallin in the lens, previously we have generated a total βA3/A1KO mouse model [15], which exhibited several phenotypic characteristics including the lack of organelles degradation in the outer cortex due to yet not fully understood mechanism of autophagy blockade. Our results showed that the autophagy blockade occurred at the fusion of lysosomes with autophagosomes to form autolysosomes, and the blockade was attributed to the absence of the βA3-crystallin in lysosomes because the crystallin has been shown to be a lysosomal resident protein in the RPE that modulates autophagy [12]. Additional lens phenotypes in the complete βA3KO mice included increased intracellular Ca^2+^ levels, and expression and activation of calpain-3 and cytoskeletal defects [15]. Although the potential role (s) of the lens phenotypic changes in the cataractogenic mechanism remains unclear, the increased Ca^2+^ levels leading to calpain activation autophagy disruption are possible suspects. To further understand molecular mechanisms of phenotypic defects associated with the absence of βA3 crystallin only in the lens, we generated the lens-specific βA3 conditional KO (βA3cKO) mouse model. This model, like our βA3 complete KO [15], also developed congenital nuclear cataracts and persistent fetal vasculature. As shown in (Fig 1), the non-expression of βA3 mRNA and immunohistochemical data confirmed the absence of βA3 at both transcriptional and translational levels and development of congenital nuclear cataract in lenses βA3cKO mice. Additionally, like previous studies [40–42], the crystallin was expressed in the retinal tissues and exhibited PFV. Together, the results suggest that the absence of βA3 crystallin in the βA3cKO lens caused the lack of cargo (organelles) clearance, and elevated Ca^2+^-induced calpain activation leading proteolysis of lens proteins, which together are likely to play an important role in cataract development.

In the present study, we focused on answering two major questions: (A) Is calpain involved in degradation of proteins in βA3cKO lenses? (B) Why fragments of proteins of microtubulin, intermediate filaments and αA-crystallin become water insoluble and whether insolubilized fragments play role in congenital cataract development in βA3cKO lenses? Finding evidence to answer the above two questions is important because our result show calpain activation and degradation and insolubilization of lens proteins occurred in βA3cKO lenses relative but not in control WT lenses. The above focus was warranted because the total βA3KO lenses also showed an elevated Ca^2+^ and calpain activation [15]. Furthermore, the present literature describes calpain-induced proteolysis of lens proteins and crystallins on an elevated Ca^2+^ levels, which plays a major role in cataract development [41–44]. Calpains (calcium-activated non-lysosomal cysteine proteases), on activation cause accelerated proteolysis of its substrates [45]. The lens contains at least four types of calpains; Calpain-1 (μ-calpain), calpain-2 (m-calpain), calpain-10 and Lp-82/Lp-85, which are lens-specific splice variants of calpain-3. Among these, Lp-82 has been identified as the major calpain that on activation causes proteolysis of lens proteins and opacity [46]. The major *in vivo* substrates of calpain-3 in the lens have been identified as α-spectrin, vimentin, and α- and β-crystallins [33,34,47–49]. Evidence also show that the uncontrolled crystallin degradation by calpain Lp82 but not by m-calpain leads insolubilization of crystallins and cataract development in rat lenses [50].

Our study shows an increased degradation and insolubilization of the lens proteins in 1-month-old βA3cKO lenses with congenital cataract compared to the age-matched lenses of wild type mice (Tables 1 to 3). The mass spectrometric results further exhibited a higher number of protein fragments in 800 rpm-spun-pellet fractions of βA3cKO lenses relative to in WT- and HET-lenses Table 1. Among these, eight of a total of eleven protein fragments were derived from of β-tubulin (from proteins with M_r_ >25 kDa), suggesting it to be a major truncation target of βA3cKO lenses. Additionally, the presence of β-tubulin fragments in the 5000-rpm-spun pellet fraction also suggested β-tubulin degradation and insolubilization of its fragments during congenital cataract development. The insolubilization of β-tubulin fragments further suggested their potential role in the cataract development in βA3cKO lenses. Although the 800-rpm-recovered pellet also contained few mitochondrial proteins, small heat shock proteins and small ubiquitin-related modifier proteins described in group II of fragments in Table 2, derived from protein of M_r_ < 25 kDa), their significance is presently unclear and being investigated.

The present study also suggests that the activation of calpain-3 was responsible for the degradation phakinin, αA-crystallin, and tubulin protein in the lenses of βA3cKO mice but not in the control lenses. The evidence presented regarding calpain-induced proteolysis of the βA3cKO lenses include: (A) In addition to β-tubulin fragments, calpain-3 fragments were also present in the 800-rpm spun-pellet fraction of βA3cKO lenses but not in WT- and HET lenses Table 3, suggesting activation and autolysis of calpain-3. This was further confirmed by the Western blot analysis with anti-calpain-3 antibody, which showed that the intensity of full-length calpain-3 was decreased with simultaneous increase of the intensity of its proteolytically cleaved fragments in the βA3cKO lenses compared to control lenses (Fig 3D). (B) Calpain-3 activity in the total protein homogenate from 1-month-old wild type- and βA3cKO-lenses was determined with a calpain substrate, N-succinyl-leu-tyr-7-amido-4-methyl-coumarin, without calcium (basal activities) and in the presence of 3 mM calcium. The βA3cKO lens homogenate exhibited a 10% increase in basal calpain activity compared to the control lenses (Fig 4A). However, in spite 20% increase in calpain-3 activity in the presence of 3 mM Ca^+2 in^ the βA3cKO lens homogenate compared to the basal calpain activity relative to the control lens homogenate, the activity was 10% lower in the presence of Ca^2+^. (Fig 4B). The explanation for the observed decrease in the Ca^2+^-induced activity was that because calpain-3 was already activated in the βA3cKO lenses in the absence of Ca^2+^ but not in the control lenses, and therefore, additional calcium did not show a major effect on hydrolysis of the substrate by the βA3cKO homogenate, but in contrast, the inactive calpain-3 got activated in the control homogenate. Alternatively, the possibility that the above commercially available calpain substrate might not be a suitable substrate for lens-specific calpain (Lp85/Lp82), and therefore, minimal difference *in vitro* calpain activity in the βA3cKO lenses relative to control lenses was seen. (C) Since calpain-3 is also its own substrate, its activation could be determined by its autolysis. Western blot analysis was used to monitor autolysis by comparing the ratio of total calpain-3 protein (the intensity of full-length calpain-3 band + intensity of cleaved fragments) to the amount of uncleaved full-length calpain-3 (intensity of full-length calpain-3 band) in βA3cKO lenses relative to control lenses. On comparison, the ratio of total calpain-3 to its uncleaved calpain-3 was 2.4 in control lenses but 1.7 in βA3cKO lenses Fig 5A and 5B. This suggested that the amount of full-length calpain-3 was decreased in βA3cKO lenses relative to WT lenses. The data further suggest activation of calpain-3 in βA3cKO lenses but not in control lenses. (D) On immunostaining of lens sections from 1-month old wild type- and βA3cKO mice with anti-calpain 3-antibody, the levels of calpain 3 staining in WT lenses was throughout the outer cortex (Fig 6A) but it was mostly localized in the outer cortex and seems to be associated with nuclei in the βA3cKO lenses (Fig 6B), suggesting possibly localization change due to calpain activation in βA3cKO lenses. Alternately, calpain 10, which is localized in lens fiber cells nuclei [51], might also be activated in the βA3cKO lenses. (E) When the wild type of lens protein homogenate was incubated at the μM level of Ca^2+^, only the calpain-3 is expected to be activated at this μM level because activation of calpain-1, calpain-2 and calpain-10 requires much higher calcium concentration of at mM levels. At the 100-μM calcium treatment, the WT lens homogenate exhibited activation of calpain-3 as judged by α-spectrin proteolysis, which was inhibited on inclusion of EGTA. In contrast, no proteolysis of β-tubulin and α-crystallin was observed by wild type lens homogenate at even μM level of Ca^2+^ in the presence or absence of EGTA. This suggested calpain-3 was activated in the wild type of lens homogenate as evidenced by α-spectrin proteolysis. The lack of the proteolysis of β-tubulin and α-crystallin could be due to a lack of lens structural changes in wild type lenses but it might be occurring in βA3cKO lenses due to cataract development. We speculate that the calpain-3 activation and proteolysis of above-described proteins occurred in βA3cKO lenses but not in the WT lenses.

The degradative loss of proteins of microtubules, intermediate filaments and αA-crystallin is relevant in congenital nuclear cataract development because tubulin along with vimentin, spectrum, actin, intermediate filaments and microfilaments etc. constitute lens cytoskeleton [52]. Cellular functions of microtubules (composed of α- and β-tubulins, both are about 50 kDa) include providing structural support, intra-cellular transport, and DNA-segregation. Dimers of α- and β-tubulins form microtubules that bind to GTP, and after microtubule assembly, the GTP bound to β-tubulin subunit hydrolyzes into GDP through inter-domain contacts, along the microtubule protofilament [53]. Tubulin dimers bound to GTP tend to assemble into microtubules, while dimers bound to GDP tend to fall apart. Alpha-crystallin might be affecting microtubule assembly by maintaining the pool of unassembled tubulin [54], whereas αB-crystallin is shown to enhance microtubule resistance to depolymerization, and maintains tubulin levels *in vivo* in muscle cells [55]. Calpain has been shown to proteolyze microtubules [56], and also calpain-mediated proteolysis, precipitation of fragments of lens crystallins and cytoskeleton proteins have been demonstrated in mice with selenite cataracts [57,58]. Because lens transparency is maintained by ordered arrangements of its components at both molecular and microscopic levels, the degradation of αA, intermediate filament proteins and β-tubulin in βA3cKO lenses would lead to instability of cytoskeleton and play a role in cataract development.

Protein aggregation is a hallmark of number of age-related diseases including the cataracts in humans and number of mouse models. Extensive literature exists about lens protein aggregation, which is caused by various factors including post-translational modifications such as deamidation, oxidation, phosphorylation, sulfonylation, mutations and protein truncation [25]. As described above, increasing evidence has emerged showing aggregation of crystallin fragments [16,17,27,28]. Three common systems for intracellular proteolysis exist (ubiquitin proteasome system, lysosomal/autophagic system and calcium-activated proteases) within the lens. Cytoplasmic organelles are degraded in the lens inner cortical region, where only ubiquitin-mediated proteasomes and calpain are operative. It is possible that in βA3cKO lenses, the lack of βA3-crystallin in fiber cells initially causes lysosomal dysfunction and thus a reduction in protein proteolysis. To compensate for this reduced proteolysis, calpain is activated, and the accelerated degradation of calpain substrate results in aggregation and insolubilization of the proteins in the nuclear region and cause nuclear cataract. Alternatively, it is also possible that the calpain activation is causing the lysosomal dysfunction as has been reported recently [59]. In addition, it is possible that because βA3 is known to be a Ca^2+^-binding protein, and therefore, its absence would release extra Ca^2+^, which in turn would activate calpain [60]. The activated calpain known to cause proteolysis of number of substrates including the heat shock chaperone hsp 70 that is localized to lysosomal membrane [61,62]. The cleavage of hsp70 by calpain causes lysosomal membrane permeabilization and therefore increase the lysosomal pH and cause inactivation of hydrolytic enzymes and defective degradation of autophagic cargo. To dissect these possibilities further, experiments are planned to examine calpain activities and protein insolubilization conducted at early time points right from the time the expression of βA3-crystallin in the lens (E13.5) to the postnatal day 5 (P5). The initial characterization of in this study will be useful in future mechanistic studies.

## Supporting information

S1 Fig (TIF)

S1 Raw images (PDF)
