# Supplementary figures and images for: Lens-specific βA3/A1-conditional knockout mice: Phenotypic characteristics and calpain activation causing protein degradation and insolubilization
Source: PLoS One. 2023 Mar 29;18(3):e0281386. doi: 10.1371/journal.pone.0281386 (PMC10057792; doi:10.1371/journal.pone.0281386)

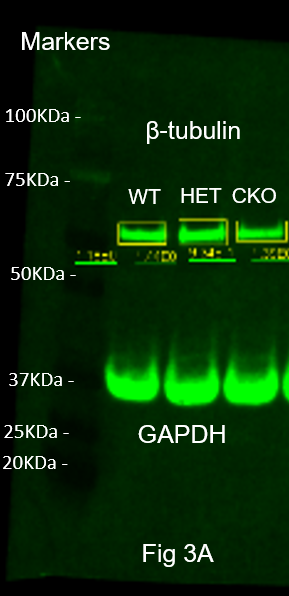

Supplement: S1 Fig — (TIF) [file pone.0281386.s001.tif]

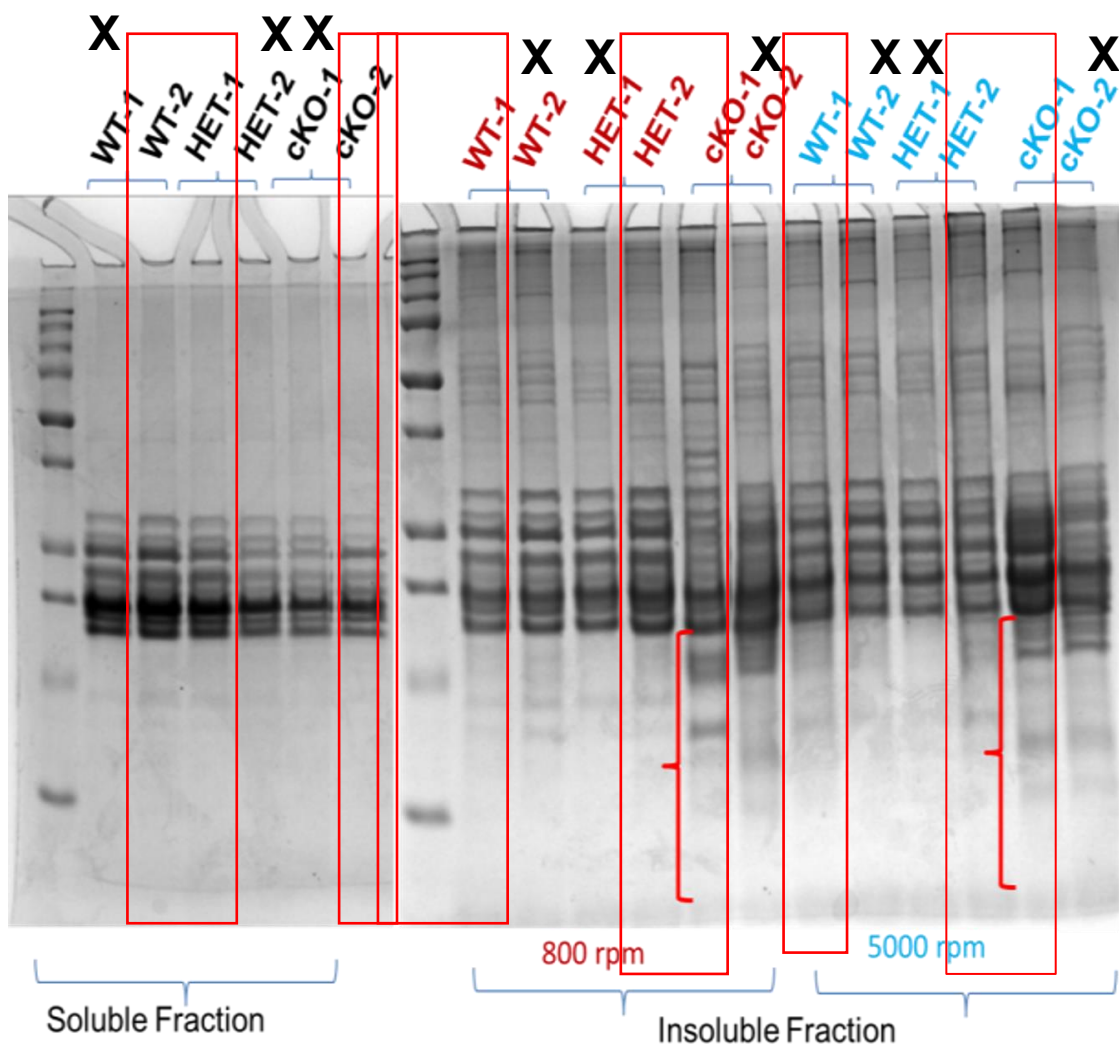

**X-** marked lanes not included in the final Figure.

**Figure 2**

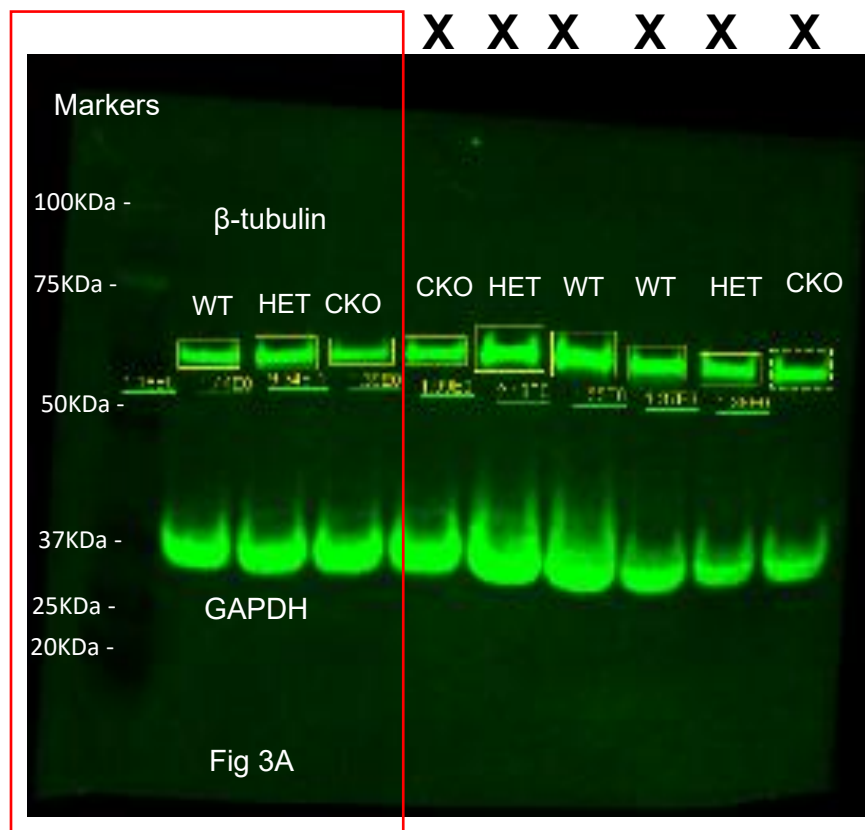

**Figure 3**

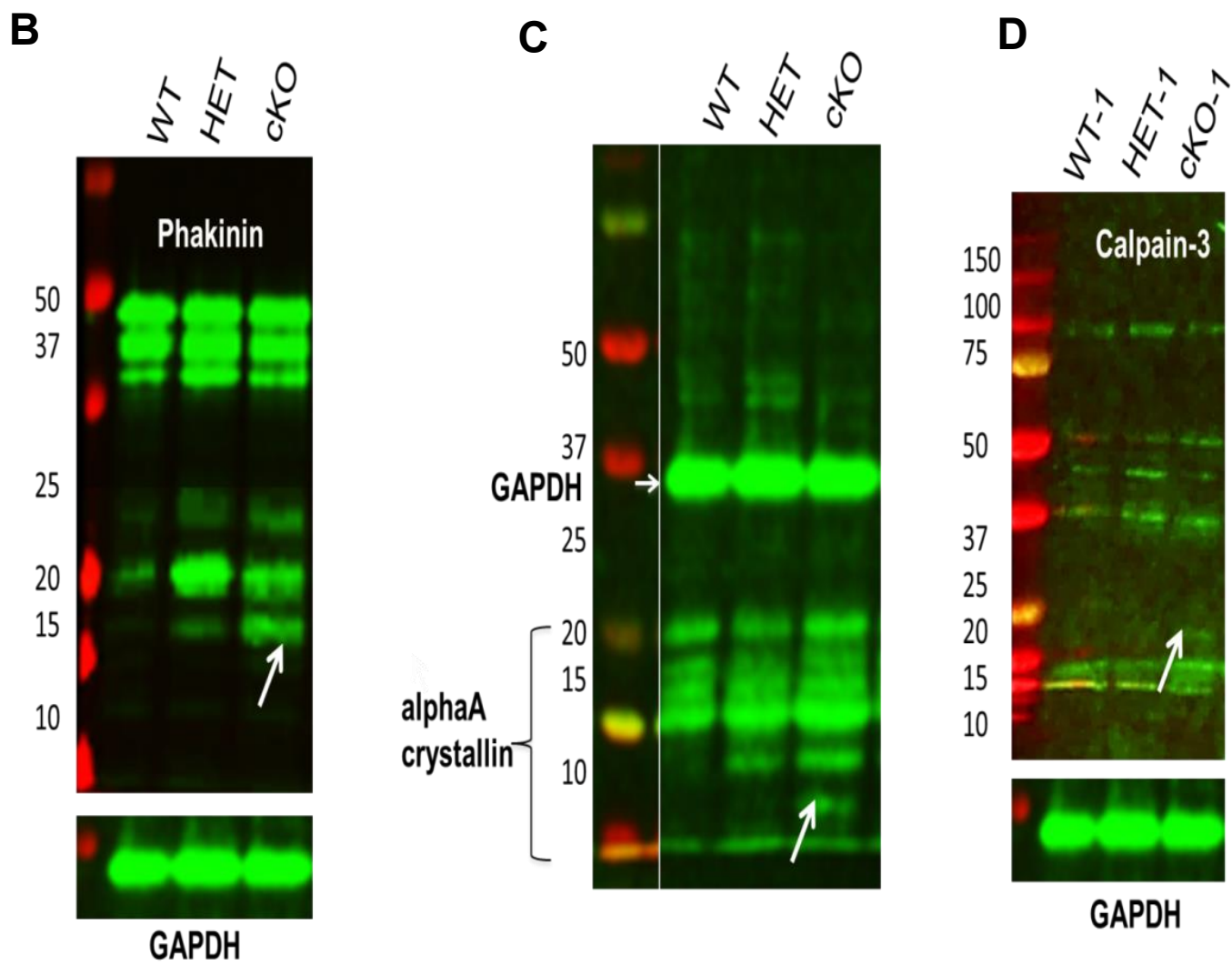

**Figure 3**

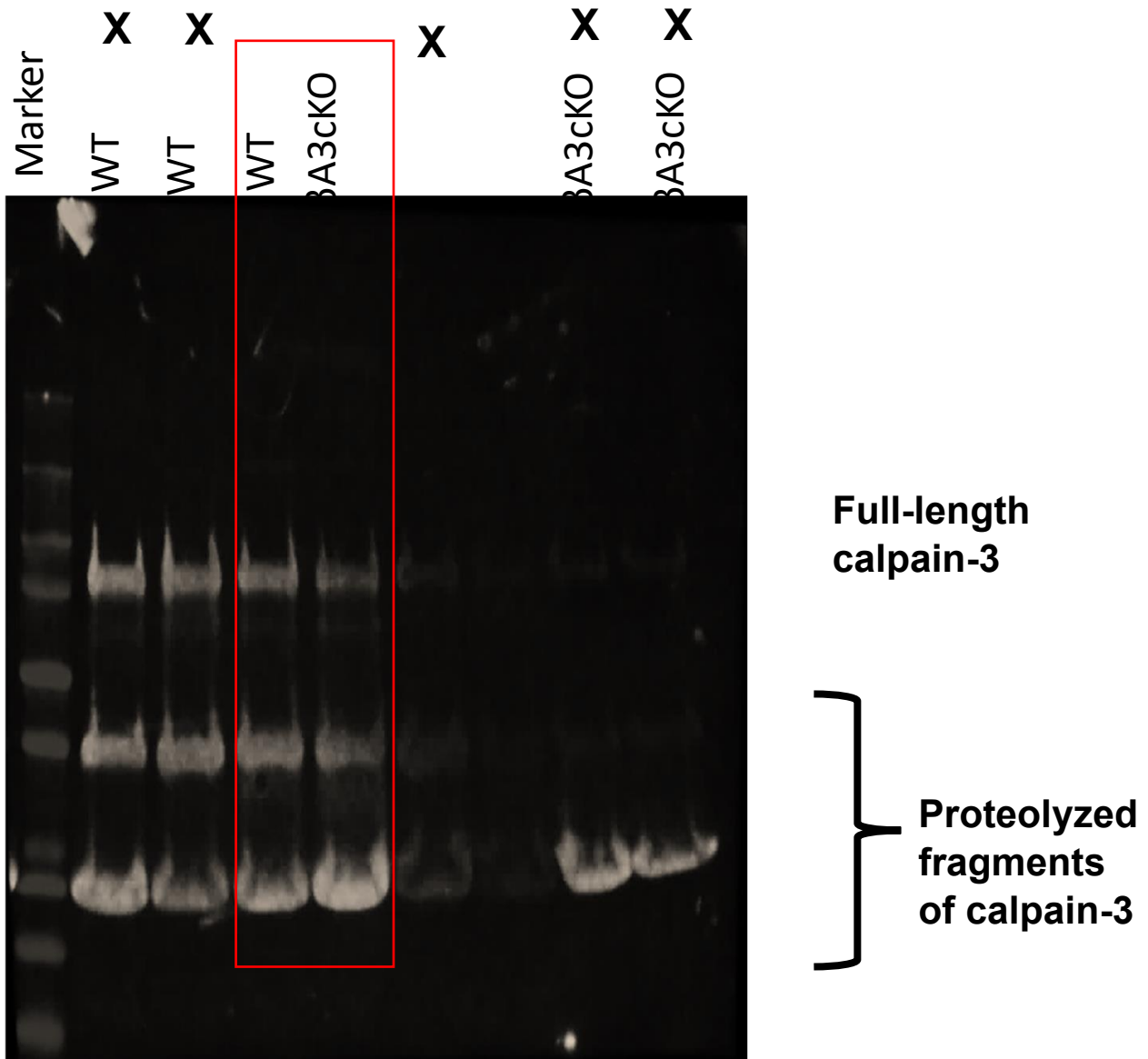

**Figure 5A**

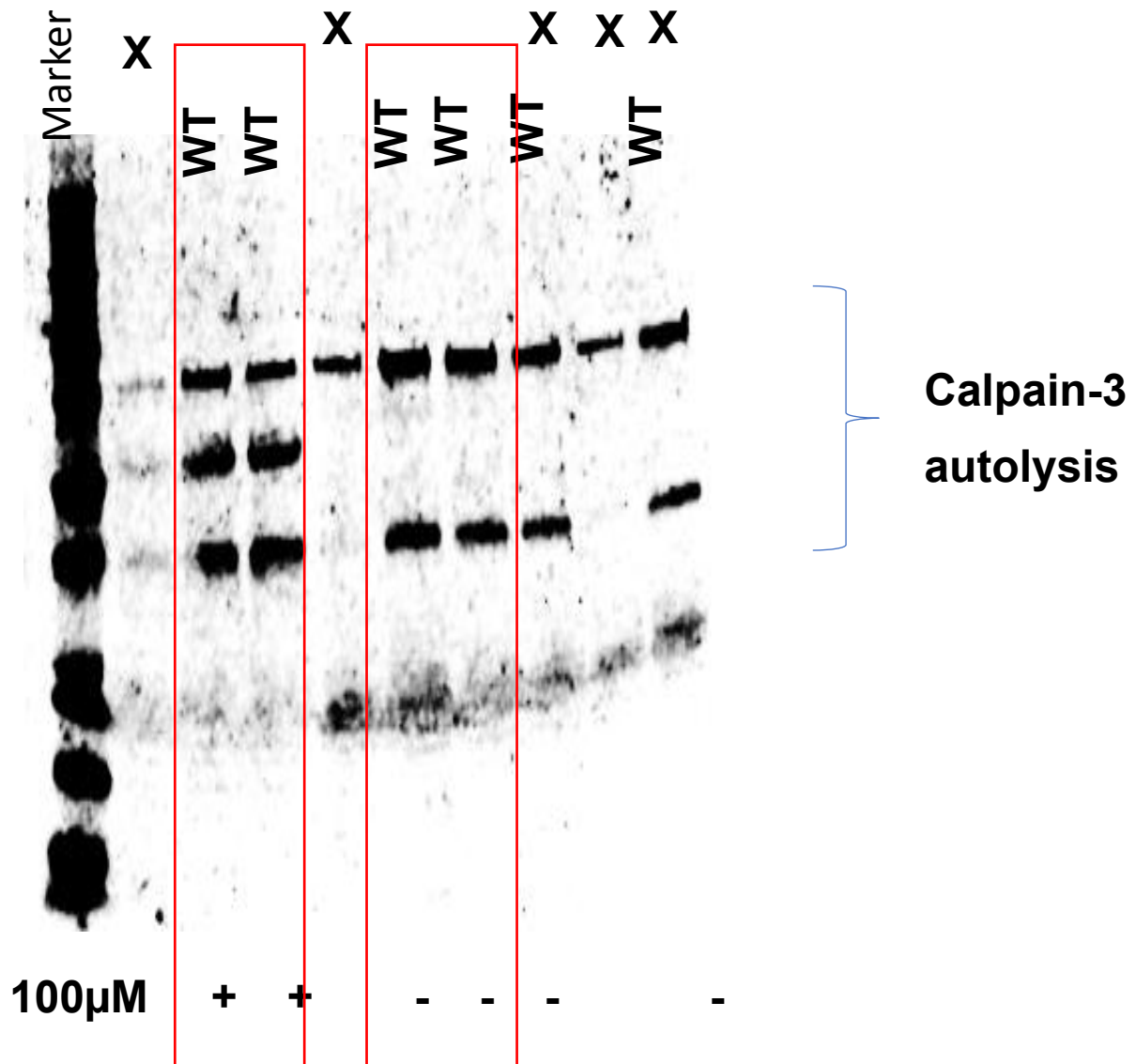

Figure 7A

Supplement: S1 Raw images — (PDF) [file pone.0281386.s002.pdf]
